# Sociocultural determinants of global mask-wearing behavior

**DOI:** 10.1073/pnas.2213525119

**Published:** 2022-10-03

**Authors:** Luojun Yang, Sara M. Constantino, Bryan T. Grenfell, Elke U. Weber, Simon A. Levin, Vítor V. Vasconcelos

**Affiliations:** ^a^Department of Ecology and Evolutionary Biology, Princeton University, Princeton, NJ 08544;; ^b^School of Public and International Affairs, Princeton University, Princeton, NJ 08544;; ^c^Andlinger Center for Energy and the Environment, Princeton University, Princeton, NJ 08544;; ^d^Department of Psychology, Northeastern University, Boston, MA 02115;; ^e^School of Public Policy and Urban Affairs, Northeastern University, Boston, MA 02115;; ^f^Department of Psychology, Princeton University, Princeton, NJ 08544;; ^g^Informatics Institute, University of Amsterdam, 1098 XH Amsterdam, The Netherlands;; ^h^Institute for Advanced Study, University of Amsterdam, 1012 GC Amsterdam, The Netherlands;; ^i^Princeton Institute for International and Regional Studies, Princeton University, Princeton, NJ 08544

**Keywords:** epidemics, public health, social norms, institutions, risk perceptions

## Abstract

Behavioral responses influence the trajectories of epidemics. During the COVID-19 pandemic, nonpharmaceutical interventions (NPIs) reduced pathogen transmission and mortality worldwide. However, despite the global pandemic threat, there was substantial cross-country variation in the adoption of protective behaviors that is not explained by disease prevalence alone. In particular, many countries show a pattern of slow initial mask adoption followed by sharp transitions to high acceptance rates. These patterns are characteristic of behaviors that depend on social norms or peer influence. We develop a game-theoretic model of mask wearing where the utility of wearing a mask depends on the perceived risk of infection, social norms, and mandates from formal institutions. In this model, increasing pathogen transmission or policy stringency can trigger social tipping points in collective mask wearing. We show that complex social dynamics can emerge from simple individual interactions and that sociocultural variables and local policies are important for recovering cross-country variation in the speed and breadth of mask adoption. These results have implications for public health policy and data collection.

On 11 March 2020, the World Health Organization (WHO) declared the spread of SARS-COV-2 (severe acute respiratory syndrome coronavirus 2) a global pandemic. Responses by countries and their residents varied tremendously, as did disease trajectories. Although there was initial ambiguity, data now suggest that mask wearing is effective in mitigating the spread of SARS-COV-2 (1), leading the WHO to publicly call for collective mask wearing “as part of a comprehensive strategy of measures to suppress transmission and save lives” (2). Despite this highly visible statement by a nonpartisan international organization, there was substantial variation in mask adoption across countries (Fig. 1*A*). Interestingly, differences in the trajectory of cases and death rates across countries do not explain the proportion of mask adopters in a population. Fig. 1*A* and *B* shows the decoupling between disease severity and the fraction of mask wearers in selected countries, as well as the presence of tipping points in some countries (e.g., the United Kingdom)—a critical rate of mask adoption that triggers a sharp and stable increase in mask wearing (3).

**Fig. 1.**
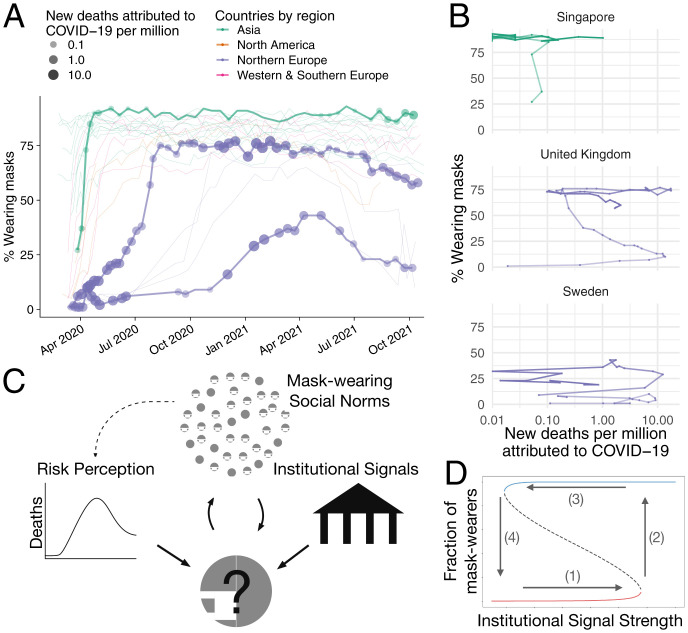
Global dynamics of mask-wearing behavior during the COVID-19 pandemic and model diagram. (*A*) Proportion of people who report wearing masks in public from March 2020 to October 2021 by country and region (12). Larger, darker circles indicate more new deaths attributed to COVID-19 (per million, 7-d smoothed) in Singapore, United Kingdom, and Sweden (thickened trajectories) (13). (*B*) Phase planes showing mask wearing vs. COVID-19 deaths in Singapore, United Kingdom, and Sweden. Darker color indicates later sample time. (*C*) Diagram of the three key elements of our mask-adoption model. (*D*) Possible tipping points in collective mask wearing. The dynamics of mask wearing are characterized by two stable equilibria (solid lines) and one unstable equilibrium (dashed line). When a population begins with low rates of mask wearing and experiences a neutral or negative institutional signal, conformity to the predominant “no mask” norm stabilizes that behavior. If there is an increase in the institutional pro–mask-wearing signal, mask wearing will slowly increase up to a critical level (red line, point 1) at which point a small increase in the institutional signal creates a behavioral cascade that results in full mask adoption (point 2). As the institutional signal wanes (point 3), the new mask-wearing norm self-sustains (blue line). A significant negative signal is required for the population to revert to non–mask-wearing equilibria (point 4).

So, what drives heterogeneity in rates of mask adoption across countries? A growing literature highlights the importance of social dynamics and norms in guiding behavior, especially for collective action problems, the adoption of novel and visible behaviors, or when risks are largely unknown and action is costly (4). The tendency to conform to social norms can create rigidity around established norms, but it can also be an engine of rapid change (3). When a minority adopts a nonnormative behavior, perhaps due to policy measures or an exogenous shock, it can spread to others in the vicinity. Once a critical mass adopts this behavior, the tendency to conform can cause the behavior to spread, resulting in the emergence of a new norm. Field studies have found that social-norm interventions effectively increase vaccine uptake (5) and mask use (6) among hesitant communities. Mathematical models highlight that social norms induce bistability in the dynamics of vaccinating behavior and could both hinder and boost vaccine uptake, depending on initial conditions (7). However, how social norms interact with top–down regulations from public health authorities remains unexplored.

Human behavior is socially and culturally embedded; individuals in different cultural contexts may respond differently to the same external conditions, policies, or information. The substantial cross-cultural variation in the tendency to conform to social norms has been called cultural “tightness” or “looseness” (8). Cultural variation in adherence to social norms is likely to affect responsiveness to measures and statements made by formal institutions to mitigate disease spread, especially when those policies are voluntary (9). Indeed, a recent study found that “tighter” countries had lower cases and deaths due to SARS-COV-2 (10). However, the mechanisms by which cultural tightness mediates population and individual response to the pandemic remain unclear (11). We contribute to this nascent literature by developing a mechanistic model of mask-wearing dynamics with sociocultural processes (Fig. 1*C* and *D*) that highlights the relationship between social norms, the stringency of policy responses, and the emergence of collective mask wearing. Modeling these social, cultural, and political features allows us to recover the diverse patterns of mask-adoption dynamics evident in the cross-country data that cannot be fully accounted for by disease dynamics alone.

## Results

**Fig. 2.**
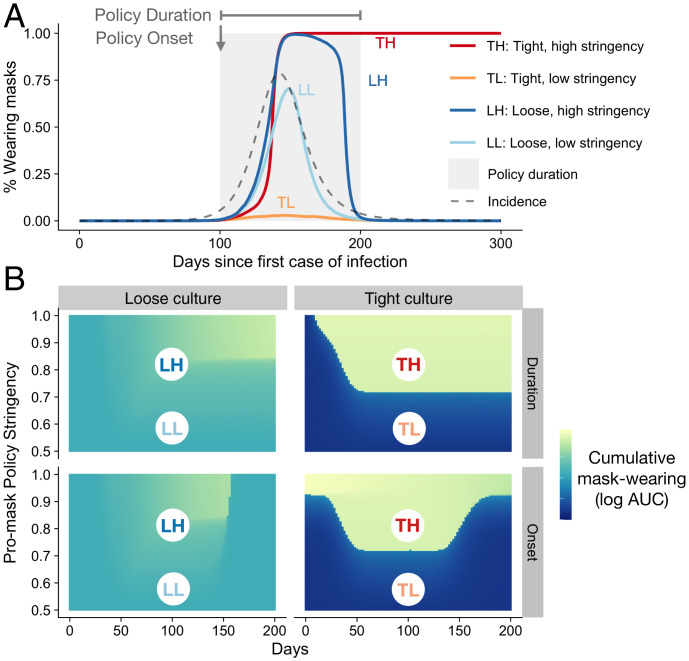
Collective dynamics of mask wearing under different social conformity and policy regimes. (*A*) Simulated epidemic (gray, dashed line) and mask-wearing dynamics under low and high levels of cultural tightness and policy stringency (solid lines). At the start of the epidemic, the policy stringency is set at neutral. As disease prevalence increases and falls, the policy response is introduced on day 100 and ends on day 200 (gray area) at low or high stringency. (*B*) Cumulative mask wearing following policies introduced at different levels of risk (onset), lasting different amounts of time (duration), and varying in strength (stringency) under low and high levels of social conformity. Color indicates the area under the mask-wearing dynamic curves (AUC) as illustrated in *A*, with lighter colors representing more mask wearing. In *Upper* row, onset of the policy is fixed at day 100 and we vary its duration. In *Lower* row, the duration of the policy is fixed at 100 days and we vary its onset. See *Materials and Methods* and *SI Appendix* for numerical implementation of the model and description of parameters.

We simulated epidemic risks resembling the first wave of the COVID-19 pandemic using a susceptible–exposed–infectious–removed–susceptible (SEIRS) model (Fig. 2*A*, dashed black line and *SI Appendix*) and introduced promask policies when the disease incidence went above a certain threshold (Fig. 2*A*, gray area). At low social conformity (loose cultures), our model predicts that collective mask wearing will follow the epidemic curve closely (Fig. 2*A*, solid blue curves). More stringent policies induce faster mask adoption during the growth phase and slower decay after the peak of the epidemic. However, the effects of policy measures in loose contexts are limited and short lived, disappearing immediately after cessation of the policy (Fig. 2*A*, solid blue curves). Increasing cultural tightness shifts the model to a bistable regime, as demonstrated in Fig. 1*D*. With low policy stringency, the existing social norm limits mask adoption and the rate of mask wearing remains low even at the peak of the epidemic (Fig. 2*A*, solid orange curve). Under high policy stringency, cultural tightness delays initial mask adoption but accelerates adoption once a critical number of adopters is reached, resulting in an abrupt shift and the emergence of a new mask-wearing norm (Fig. 2*A*, solid red curve). The tighter the culture is, the more likely collective mask wearing persists even following reductions in contagion risks and relaxation of policy measures (Fig. 2*B*).

Importantly for public health policy, the tendency to conform to social cues interacts with features of the policy response. In Fig. 2*B*, we vary the stringency, onset, and duration of the policy and show the cumulative mask wearing (quantified by the area under the mask-wearing dynamic curve). Low cultural tightness dampens the effects of policy responses (Fig. 2*B*, *Left* column). Under high conformity, collective mask wearing responds nonlinearly to increases in policy stringency, rising sharply once policy stringency crosses a certain threshold (Fig. 2*B*, *Right* column). Thus, the success of an intervention is sensitive to the timing of its introduction, its duration, and its stringency, as well as the local tendency to conform to social norms. If a signal is sufficiently strong to initiate a behavioral cascade, early interventions are preferable for sustaining mask-adoption rates. If it does not initiate behavioral cascades alone, it may be more effective to introduce mask-related policies when disease risk is also increasing or high. Unsurprisingly, more stringent and longer interventions are consistently more effective.

## Discussion

The diversity of responses by policymakers and the public to the COVID-19 pandemic has ushered in research focused on the psychological, social, and cultural antecedents of social distancing and mask wearing. We expand on this literature with a model of mask-wearing decisions incorporating cultural differences in conformity to social norms and in policy measures of formal institutions. These features can produce resistance to mask wearing in some contexts; rapid adoption in others; and, importantly, the social tipping dynamics evident in cross-country mask-adoption data. The existence and quantitative aspects of tipping points in the system are known to depend on individual parameter distributions (especially their modality) (14). The model provides insights about how cultural factors and policy responses interact; for instance, the effects of early “anti-”mask signals, such as the initial declaration by the WHO, can be difficult to reverse in “tight” societies and may require strong countervailing signals from formal institutions.

This study identifies urgent priorities for theory development and data collection for pandemic intervention. Although a growing number of epidemiological models of SARS-COV-2 transmission recognize the importance of human behavior, the mechanisms of how behavioral responses are mediated by sociocultural contexts remain understudied. Our modeling framework suggests several avenues for future models. First, incorporating sociocultural considerations in coupled disease–behavior models could assess the long-term effect of behavioral feedback on disease transmission (15). Second, parallel efforts to develop cognitive models of risk and social norm perceptions, including demographic dependencies, would help address within-population behavioral heterogeneities. Furthermore, social learning and network structure or the alignment between behaviors and social identities are likely to impact the dynamics described here (16). Incorporating these processes in extended models could aid analysis of heterogeneity in collective mask wearing within countries as well as the evolution of more complex behavioral strategies such as vaccination. Modeling efforts must evolve in tandem with data collection that measures cross-country sociocultural variables, their distribution, and the effect of peer influence on mask adoption, e.g., using contact-tracing apps. Additionally, countries have different histories with the use of personally protective measures to mitigate the spread of respiratory illnesses. Taking these histories and latent social norms toward nonpharmaceutical interventions into account may offer insights into the efficacy and design of policies aimed at shaping collective behaviors across contexts.

Our findings contribute to the understanding of the relationship between disease severity, social dynamics, and institutional responses in different cultural contexts. Mask wearing and other nonpharmaceutical interventions (NPIs) are important control measures especially for overdispersed respiratory infections with high breakthrough infection rates that resemble SARS-COV-2. The focus on social norms is likely relevant for collective action in the face of other crises. This work, and suggested extensions, can support the design of preventive interventions as opposed to the reactive ones that have characterized the first 2 years of the COVID-19 pandemic.

## Materials and Methods

### Data.

Cross-country self-reported mask-wearing rates are from YouGov survey data and the COVID-19 Public Monitor (https://today.yougov.com/covid-19). Epidemiological data are from Our World in Data (https://ourworldindata.org/covid-deaths). Source code data are available on GitHub (https://github.com/luojun-yang/mask_dynamics).

### Threshold Model of Social Norm Dynamics.

In our model, individuals consider the social and personal utility of wearing a mask, looking to maximize it. Inputs to this decision are 1) net direct benefit of mask wearing, which depends on the difference between the perceived reduction in contagion risk, here taken to be proportional to the disease incidence, and a fixed cost (e.g., monetary cost, physical discomfort, inconvenience of mask wearing); 2) social net benefits from conforming to the descriptive social norm, here taken to be proportional to the fraction of the population that adopts the behavior; and 3) benefits from adhering to (or costs to deviating from) the injunctive norm, here taken to be mandates or statements by formal institutions (Fig. 1*C*), which are dynamic and exogeneous to the system. Whereas input 1 depends on individual perceptions of infection probability, inputs 2 and 3 depend on an individual’s tendency to conform. Distributions of individual-level parameters are centered around different country-specific measures of risk and conformity. Based on these aspects, agents synchronously update their behavior to maximize their utility (details in *SI Appendix*). We implement an agent-based model to simulate the behavioral dynamics.

Our behavioral model can be seen as an extension of Gavrilets’ model of injunctive social norms (14), with the following key modifications: 1) dynamic institutional signals, 2) cultural specific conformity (tightness), and 3) disease dynamics as external forcing.

## Supplementary Material

Supplementary File

## Data Availability

Source code data are available in the GitHub Repository (https://github.com/luojun-yang/mask_dynamics) (17). Previously published data were used for this work (12, 13).

## References

[1] J. Howard ., An evidence review of face masks against COVID-19. Proc. Natl. Acad. Sci. U.S.A. 118, e2014564118 (2021). 33431650 10.1073/pnas.2014564118 PMC7848583

[2] World Health Organization, When and how to use masks. https://www.who.int/emergencies/diseases/novel-coronavirus-2019/advice-for-public/when-and-how-to-use-masks. Accessed 26 September 2022.

[3] M. Granovetter, Threshold models of collective behavior. Am. J. Sociol. 83, 1420–1443 (1978).

[4] K. Nyborg ., Social norms as solutions. Science 354, 42–43 (2016). 27846488 10.1126/science.aaf8317

[5] A. Karing, Social Signaling and Childhood Immunization: A Field Experiment in Sierra Leone (University of California, Berkeley, CA, 2018). 10.1093/qje/qjae025 PMC11461810 39391631

[6] J. Abaluck ., Impact of community masking on COVID-19: A cluster-randomized trial in Bangladesh. Science 375, eabi9069 (2021). 10.1126/science.abi9069 PMC9036942 34855513

[7] T. Oraby, V. Thampi, C. T. Bauch, The influence of social norms on the dynamics of vaccinating behaviour for paediatric infectious diseases. Proc. Biol. Sci. 281, 20133172 (2014). 24523276 10.1098/rspb.2013.3172 PMC4078885

[8] M. J. Gelfand ., Differences between tight and loose cultures: A 33-nation study. Science 332, 1100–1104 (2011). 21617077 10.1126/science.1197754

[9] C. Betsch ., Social and behavioral consequences of mask policies during the COVID-19 pandemic. Proc. Natl. Acad. Sci. U.S.A. 117, 21851–21853 (2020). 32820078 10.1073/pnas.2011674117 PMC7486713

[10] M. J. Gelfand ., The relationship between cultural tightness–looseness and COVID-19 cases and deaths: A global analysis. Lancet Planet. Health 5, e135–e144 (2021). 33524310 10.1016/S2542-5196(20)30301-6 PMC7946418

[11] J. G. Lu, P. Jin, A. S. English, Collectivism predicts mask use during COVID-19. Proc. Natl. Acad. Sci. U.S.A. 118, e2021793118 (2021). 34016707 10.1073/pnas.2021793118 PMC8201834

[12] S. P. Jones; Imperial College London Big Data Analytical Unit, *YouGov Plc., Imperial College London YouGov Covid Data Hub, v1.0* (2020).

[13] E. Dong, H. Du, L. Gardner, An interactive web-based dashboard to track COVID-19 in real time. Lancet Infect. Dis. 20, 533–534 (2020). 32087114 10.1016/S1473-3099(20)30120-1 PMC7159018

[14] S. Gavrilets, The dynamics of injunctive social norms. Evol. Hum. Sci. 2, e60 (2020). 10.1017/ehs.2020.58 PMC10427483 37588350

[15] Z. Qiu ., Understanding the coevolution of mask wearing and epidemics: A network perspective. Proc. Natl. Acad. Sci. U.S.A. 119, e2123355119 (2022). 35733262 10.1073/pnas.2123355119 PMC9245665

[16] V. V. Vasconcelos ., Segregation and clustering of preferences erode socially beneficial coordination. Proc. Natl. Acad. Sci. U.S.A. 118, e2102153118 (2021). 34876514 10.1073/pnas.2102153118 PMC8685719

[17] L. Yang, mask_dynamics. GitHub. https://github.com/luojun-yang/mask_dynamics. Deposited 14 September 2022.

