# Supplementary material for: Sociocultural determinants of global mask-wearing behavior
Source: Proc Natl Acad Sci U S A. 2022 Oct 3;119(41):e2213525119. doi: 10.1073/pnas.2213525119 (PMC9565043; doi:10.1073/pnas.2213525119)
Supplement: Supplementary File [file pnas.2213525119.sapp.pdf]

PNAS
www.pnas.org

Supplementary Information for

Sociocultural determinants of global mask-wearing behavior

Luojun Yang, Sara M. Constantino, Bryan T. Grenfell, Elke U. Weber, Simon A. Levin, Vítor V. Vasconcelos

Luojun Yang, Vítor V. Vasconcelos
E-mail: luojuny@princeton.edu, v.v.vasconcelos@uva.nl

This PDF file includes:

Supplementary text
SI References

Supporting Information Text

Behavioral model. We consider a population of Z individuals. The actions of individual i in culture C depend on (a) the perceived net benefits of wearing a mask, b_i, (b) the conformity level, w_C, which modulates the impact of (c) d_i, the individual-specific weight on alignment with the descriptive norm (here, y_i, the fraction of people that individual i sees wearing masks)(1) and (d) the stringency of the mask-wearing signal, s_C, also modulated by (e) p_i, an individual-specific weight on alignment with the formal institutional signal (trust). Thus, we write the utility of wearing a mask (U_{ic}^M) or not (U_{ic}^N), respectively, as

$$\begin{aligned} U_{ic}^M &= b_i + w_C\left(d_i y_i + p_i s_C \right) \\ U_{ic}^N &= \underbrace{}_{\text{i) net benefit of mask wearing}} \; w_C\left(\underbrace{d_i(1-y_i)}_{\text{ii) alignment with descriptive norm}} + \underbrace{p_i(1-s_C)}_{\text{iii) alignment with institutional signal}} \right), \end{aligned} \quad [1]$$

Benefit b_i depends on the probability of infection and is assumed to be proportional to a population's disease incidence I. For each culture, w_C correlates with the tightness-looseness score that quantifies tendency to conform to perceived social norms. For simplicity, we drop the C from our notation as we assume non-interacting cultures. When deciding whether to update their behaviors, individuals consider the marginal gains of changing strategy, $\Delta U_i^{N\to M} = U_i^M - U_i^N = -\Delta U_i^{M\to N}$, where the last equality assumes no action-dependent biases and no change in evaluation of payoffs or personal norms from individual i changing her strategy. The sign of $\Delta U_i^{N\to M}$ determines whether i wants to wear a mask. Everyone has a fixed probability per unit time of considering whether to change their behavior (μ).

$\Delta U_i^{N\to M}$ is exogenously forced by the stringency of mask-wearing signal (s) and disease incidence (I) and, thus, time dependent, while the descriptive norm (y_i) changes endogenously (though we omit such time dependency from our notation). Since $\Delta U_i^{N\to M}$ increases (sensu lato) with y_i, there is at most one (real) threshold value, T_i, of y_i above which individual i will choose M and below which they will choose N. In this case,

$$T_i = \frac{1}{2} + \frac{p_i(1-2s)}{2d_i} - \frac{b_i}{2wd_i}. \quad [2]$$

Thus, we can map the distribution of individual-level parameters and the institutional norms (the ingredients of Eq.(1)) into a distribution of threshold values, $\rho[T]$.

Transmission-behavior model. We consider an extension of the behavioral model described above, where risk perception is derived from a susceptible-exposed-infectious-removed (SEIRS) model (2).

$$\begin{aligned} \frac{dS}{dt} &= -\beta SI + \delta R \\ \frac{dE}{dt} &= \beta SI - \tau E \\ \frac{dI}{dt} &= \tau E - \gamma I \\ \frac{dR}{dt} &= \gamma I - \delta R \end{aligned}$$

In this model, S, E, I, R denote the proportions of susceptible, exposed, infectious, and recovered individuals, respectively, given transmission rate β, immune waning rate δ, transition to infectious rate τ, recovery rate γ. We do not include asymptomatic transmission, age structure, behavioral feedback, and other complexities of SARS-COV-2 transmission, as we focus on collective behavioral response to the first wave of transmission.

Numerical simulations. Based on the analytical formulations, we develop an agent-based model to simulate mask-wearing behavior in a well-mixed population of 10000 individuals. First, daily disease incidence (I) is simulated from the SEIRS model described above. Then the population mean of perceived risk reduction from wearing a mask ($\bar{r}$) is taken to be proportional to simulated incidence, $\bar{r} = \alpha I$, and the individual benefit from perceived risk reduction follows a Gamma distribution $r_i \sim \Gamma(\bar{r}^2/\sigma, \sigma/\bar{r})$. The net benefit of wearing a mask for individual i is $b_i = -\kappa + r_i$, where κ denotes a fixed cost of mask-wearing (monetary and negative impact on social interactions). In all simulations, we set $\kappa = 0.1$, $\alpha = 11$, which guarantee a response to the fraction of infected, and the remaining parameters relate to the properties of SARS-COV-2 transmission, namely, transmission rate $\beta = 0.44$ days^{-1} (corresponding to $R_0 = 2.2$), mean incubation period $1/\tau = 5$ days, mean infectious period $1/\gamma = 5$ days, and mean duration of immunity $1/\delta = 365$ days. Second, individual weights d_i and p_i are drawn from Gamma distributions $\Gamma(\bar{d}^2/\sigma, \sigma/\bar{d})$ and $\Gamma(\bar{p}^2/\sigma, \sigma/\bar{p})$, respectively. Note that we choose Gamma distribution to capture individual heterogeneity in risk perception (r_i), individual weights on conformity to descriptive social norm (d_i) and trust in institution (p_i) as one of the simplest tunable models for non-negative parameters. Extensions to other unimodal, non-negative distributions (e.g., lognormal) do not change the main results qualitatively. However, including, e.g., bimodal distributions can affect the results by giving rise to additional intermediate equilibria. We would also like to highlight that even though the distribution of parameters is a Gamma distribution, the threshold distribution for behavioral change ($\rho[T]$) is not. It is instead a combination of sums and products of parameter distributions. Last, at probability $\mu = 0.3$, agents update

their behavior synchronously at time t to the one that increases their utility based on observed behavior of neighbors and policy stringency at time $t-1$.

For simulations under different levels of cultural tightness and policy stringency (Figure 2), we set $w = 0.1$ for loose culture and $w = 0.8$ for tight culture, $s = 0.55$ for low stringency and $s = 0.8$ for high stringency. When institutional signaling is neutral, $s = 0.5$.

References

1. S Gavrilets, PJ Richerson, Collective action and the evolution of social norm internalization. *Proc. Natl. Acad. Sci.* **114**, 6068–6073 (2017).
2. SM Kissler, C Tedijanto, E Goldstein, YH Grad, M Lipsitch, Projecting the transmission dynamics of sars-cov-2 through the postpandemic period. *Science* **368**, 860–868 (2020).
